# Mechanical and Shape Recovery Characterization of MWCNTs/HNTs-Reinforced Thermal-Responsive Shape-Memory Polymer Nanocomposites

**DOI:** 10.3390/polym15030710

**Published:** 2023-01-31

**Authors:** Sivanagaraju Namathoti, Manikanta Ravindra Kumar Vakkalagadda

**Affiliations:** School of Mechanical Engineering, VIT-AP University, Amaravati 522237, Andhra Pradesh, India

**Keywords:** shape-memory polymer, multiwalled carbon nanotubes, halloysite nanotubes, shape recovery

## Abstract

Mechanical and shape recovery characteristics of thermal-responsive shape-memory polyurethane (SMPU) reinforced with two types of reinforcements, multiwalled carbon nanotubes (MWCNTs) and halloysite nanotubes (HNTs), were studied in the present research work. Three weight percentages of reinforcement (0, 0.5 and 1%) in the SMPU matrix were considered, and the required composite specimens were obtained through injection moulding. Tensile, flexural, impact and shape recovery behaviours were studied experimentally. Further, flexural tests were performed for multiple cycles to understand the specimens’ flexural strength variation after shape recovery. The concentration of both reinforcements (MWCNTs and HNTs) considered in the present study significantly improved mechanical properties and shape recovery.

## 1. Introduction

Polymers have become one of the most favoured materials for engineering applications due to their low cost, reliability, reproducibility and simplicity of processing. As a result of the development of sophisticated materials for possible applications, there has been a significant increase in research using polymer composites during the last several decades. Shape-memory polymers (SMPs) are an innovative class of materials that memorize their shape/configuration and return to their original shape in response to external stimuli [1]. SMPs are gaining popularity due to their low density, high percentage of shape recoverability and ease of processing [2]. SMPs have an enormous potential for usage in various applications, including medical devices [3], sensors ([4,5,6]) and actuators ([7,8]). SMPs could be thermoplastics or thermosets, and thermoplastic SMPs are more popular due to their ability to be melted and reshaped several times. SMPs may be triggered by external stimuli such as heat [9], electricity [10], light [11], magnetism [12], water [13], microwaves [14], pressure [15], chemical solvents [16] and sound. SMPs that respond to heat (as a stimulus) are called thermal- or thermoresponsive SMPs.

SMPs used to have a permanent shape and a temporary programmed shape. A thermoresponsive thermoplastic SMPs permanent form is assigned during the manufacturing process. This procedure may include traditional polymer processing techniques such as extrusion or injection moulding. To assign a temporary shape, the SMP is deformed to the desired shape using an external physical constraint when the SMP is heated above its glass transition temperature (Tg). It is then cooled to temperatures below Tg, while the constraint is still applied to fix the desired temporary shape. After the component is cooled below Tg, the physical constraint is eliminated, and the temporary shape is attained. The sample regains its permanent shape after being reheated above Tg. Once retrieved, the sample retains its permanent shape until further programming is performed. Injection moulding is a low-cost manufacturing technique for producing various components using thermoplastic polymers.

The shape-memory polymers’ ability to recover their permanent shape on one side and the high efficiency of manufacturing using injection moulding on the other side brought a combination of ease of processing and improved properties to SMPs. Extensive research has been performed various shape-memory polymers and their composites with various external stimuli such as temperature, light and magnetism. The mechanical properties of carbon and glass-fibre-reinforced hybrid shape-memory polymer composites were studied by [17] and the authors obtained the flexural modulus as a function of the mixing ratios of fibre reinforcement and temperature. The thermomechanical and electroactive properties of styrene-based SMP with conductive elastic fabric were studied by [18] and the authors observed a stability in the results even after 50 cycles at 40% tensile strain. The influence of temperature, strain and rate on the shape recovery of thermoplastic polyurethane were investigated by [19] using injection moulding. It was observed that all the above three parameters were significantly affecting the shape recovery. Further, a higher shape recovery was observed in a thermomechanical analysis at higher strain rates.

SMP reinforced with unidirectional carbon fibres developed by [20] exhibited variable stiffness characteristics. Thermoresponsive SMPU reinforced with graphene nanoplatelets and MWCNTs, which are responsive to microwave irradiation, were studied by [21]. One-percent graphene-nanoplatelets-reinforced composite exhibited the highest shape recovery of 90% under microwave irradiation. Automatic opening and closing mechanisms for dynamic shading devices using shape-memory polymer, which respond to the temperature as an external stimulus, were developed by [22]. The effect of moisture content on the variation in the glass transition temperatures of SMP reinforced with graphene nanoplatelets (for microwave irradiation as an external stimulus) was studied by [23]. A decrease in the glass transition temperature was observed with an increase in moisture content. The thermomechanical and shape recovery characteristics of a shape-memory polyurethane silicon carbide and clay hybrid composite were studied by [24]. Results showed that the hybrid composite exhibited better thermal stability and improved tensile strength. The authors in [25] tried to improve the mechanical properties of microwave-induced thermoresponsive shape-memory thermoplastic polymer reinforced with MWCNTs and reported that the mechanical and shape recovery improved by increasing the percentage of MWCNTs in the shape-memory polymer matrix. Further, their study indicated a clear increase in the glass transition temperature with the increase of the reinforcement. The pure SMPU specimen exhibited a tensile strength of 58 MPa, but when reinforced with MWCNTs with 0.5, 1 and 1.5 wt%, the tensile strength results were 61 MPa, 66 MPa and 69 MPa, respectively. The performance of joule heat-responsive SMP polymer composite was studied by [26] and the authors obtained the optimum actuation voltage for a better response.

The programmable behaviour of 4D-printed shape-memory polymer composites (polylactic acid as the matrix material and carbon nanotubes as reinforcement) was studied by [27]. The results showed clearly that there was an increase in the glass transition temperature with the increase in the percentage of carbon nanotubes reinforcement. The shape-memory behaviour and mechanical properties of shape-memory thermoset materials with graphite as reinforcement was studied by [28] and the results showed that the glass transition temperature increased with the increase in the weight percentage of graphite reinforcement. The results of [29] showed that with an increase in weight percentage of CNTs and HNTs, the tensile and flexural strengths were increased. Further, specimens reinforced with HNTs exhibited a higher flexural strength than that of CNTs. Experiments of [30] showed the weight percentage of HNTs significantly improved the tensile and flexural properties of polyester resin. The addition of MWCNTs significantly increased the tensile properties of PMMA [31].

A few earlier reports only presented the mechanical and shape recovery characteristics of SMPU reinforced with various carbon-based reinforcements. The present study deals with the mechanical (tensile, flexural and impact) and shape recovery characterization of MWCNTs/HNTs-reinforced thermoplastic SMPU fabricated using injection moulding. The effect of the percentage of reinforcement on mechanical and shape recovery characteristics was studied with MWCNTs and HNTs. Further, for the first time, the flexural behaviour of pure and reinforced specimens for multiple cycles of the flexural test is presented to understand the actual behaviour of these composites. Further, to address the behaviour of SMPU, a flexural test (a three-point bending test) for multiple cycles was performed to understand the material behaviour under flexural loading after complete shape recovery.

## 2. Materials, Experimental and Characterization Procedure

### 2.1. Shape-Memory Polymer and Reinforcements

A thermal-responsive shape-memory polyurethane (MM-5520 and ether type) with a glass transition temperature of 55 °C was used as a polymer matrix material. Two types of reinforcements were used for the present study: (i) multiwalled carbon nanotubes (MWCNTs) of 99% purity, a diameter of 5–20 nm and a length of 10 µm; (ii) halloysite nanotubes (HNTs) of 99.9% purity, a diameter of 15 nm and a length of 1–15 µm.

### 2.2. Preparation of Specimens for Various Characterizations

**Figure 1:**
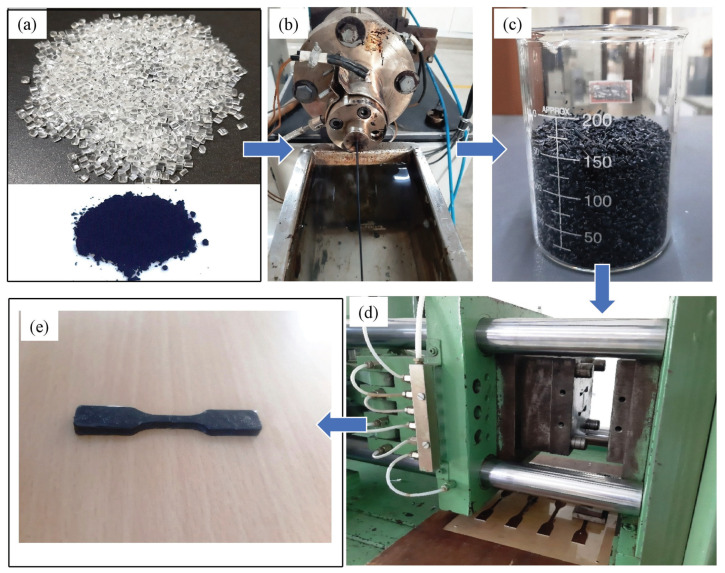
Steps involved in the fabrication of a specimen using injection moulding. (**a**) SMPU pellets, MWCNTs reinforcements, (**b**) obtention of composite filaments (SMPU and reinforcements) from the twin screw extruder, (**c**) pellets prepared from composite filaments which serve as input to the injection moulding, (**d**) injection moulding die and (**e**) tensile test specimen prepared from pure SMPU and composite material (SMPU and reinforcements).

Shape-memory polymer material obtained in pellet form from the supplier was used as a matrix material. Both reinforcements (MWCNTs and HNTs) in powder form were used to prepare reinforced polymer composite samples from injection moulding. Shape-memory polymer pellets (SMPPs) and reinforcements were dried for 3–4 h in a vacuum oven at 80 °C to eliminate carbonaceous contaminations and moisture. A twin screw extruder was used to obtain the preblend of the nanocomposite of SMPPs and reinforcements. A corotating 25 mm parallel twin screw lab extruder from Aasabi Machinery Pvt. Ltd. (Aasabi/25TS/CO/300/30) was used to obtain the preblends of the required polymer and reinforcement. Three percentages of reinforcement, 0%, 0.5% and 1% by weight, were used for this study. The filaments (from the twin screw extruder) of the required combination of matrix and reinforcement were used as input for the injection moulding. An injection moulding machine (semiautomatic from Deesha Impex-IM-D30) was used to create tensile and flexural sample specimens. Figure 1 shows the complete processing of the polymer composite (SMPU reinforced with MWCNTs) and how we obtained the required specimens. During injection moulding, the temperature profile, such as injection pressure and plasticizing temperatures, were set depending upon the manufacturer’s guidelines. The melting temperature was set to 190–195 °C at an injection pressure of 60 bar.

### 2.3. Differential Scanning Calorimetry (DSC) Test

DSC analyses were performed on all specimens (pure and reinforced SMPU) to understand the variation in glass transition (Tg) and melting temperatures (Tm). Experiments were carried out on a simultaneous thermal analyser (SAT 8000 from PerkinElmer) at a temperature range of 25 °C to 250 °C, with a heating rate of 20 °C/min and with an approximate sample weight of 25 mg, where the heating of the apparatus started from the room temperature, which was 25 °C. Tg and Tm were evaluated by obtaining the inflection point (steepest point) identified on the steps observed in the respective zones of the heat flow curve through the evaluation of the slope.

### 2.4. Tensile Test

**Figure 2:**
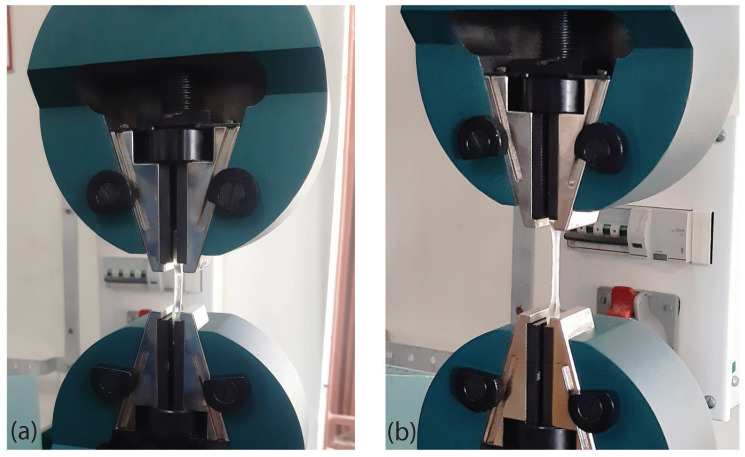
(**a**) Initial and (**b**) deformed configurations of a pure SMPU specimen while performing tensile test.

Tensile tests were performed using wedge clamps tensile testing equipment (Tinius Olsen H10 KL). The ASTM D638 (Type-V) samples were used to perform the test, and all the tensile tests were performed at room temperature (27 °C) with a crosshead displacement of 2 mm/min. Figure 2 shows the initial and deformed configurations of various samples considered.

### 2.5. Flexural Test

**Figure 3:**
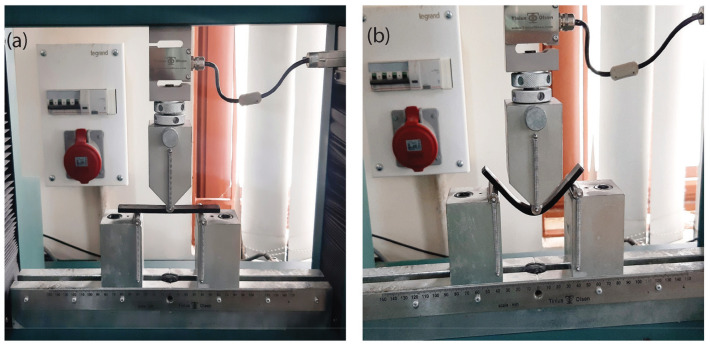
(**a**) Initial and (**b**) deformed configurations of SMPU and MWCTs composite specimen while performing the flexural test.

Flexural tests were carried out using tensile testing equipment (Tinius Olsen H10KL) in three-point bending mode with a gauge length of 80 mm. The ASTM D790 with dimensions of 125 × 15 × 5 mm^3^ was used to perform the flexural test and all the flexural tests were performed at (25 °C) room temperature with a crosshead displacement of 1 mm/min. The flexural stress and strain values were monitored throughout the test. Figure 3 shows the specimen’s initial and deformed configuration (30 mm vertical punch displacement) during the three-point bending test. All mechanical tests were as per ASTM standards and each experiment was repeated five times.

### 2.6. Shape Recovery Test and Flexural Tests for Multiple Cycles

**Figure 4:**
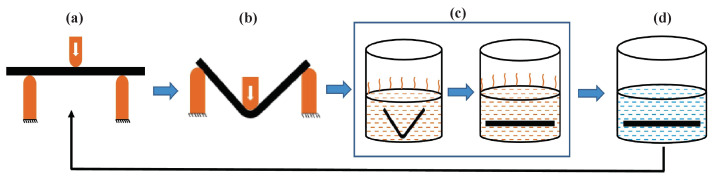
Schematic of flexural test performed for repeated cycles. (**a**) Initial position of the sample for performing the flexural (3-point bending) test, (**b**) deformed mode of the specimen at the end of the flexural test, (**c**) shape recovery by heating above Tg, (**d**) cooling and shape fixing of the sample at room temperature and performing the flexural test on the sample once again.

After complete deformation at the end of the flexural test, the sample(s) were heated to above the glass transition temperature (in a water bath at 80 °C) to regain their permanent shape. The corresponding time required for complete shape recovery was noted for all considered cases, along with the angle of the deformed sample recorded during the shape recovery. Equation (1) shows the expression for a percentage of shape recovery during the test, where $\Theta_{ud}$ is the angle in the undeformed or permanent shape condition as shown in Figure 4a and $\Theta_{d}$ is the angle at the deformed position as shown in Figure 4b. After regaining the permanent shape, samples were cooled to room temperature, and flexural test(s) were carried out again. These experiments were carried out for multiple cycles for all samples considered in this study. Figure 4 shows the schematic of a cycle, where a sample is deformed in the flexural test and the permanent shape is regained through water bath heating and the flexural test is performed once again.

(1) $$R\%=\left(1-\frac{\Theta_{ud}-\Theta_{d}}{\Theta_{ud}}\right)\times 100\%$$

### 2.7. Impact Test

The Charpy impact tests were carried out using a computerized Izod Charpy impact tester (LT-160 from International Equipments) to determine the impact strength of a notched specimen (for pure SMPU and reinforced with MWCNTs and HNTs) while breaking under impact load. The Charpy impact test specimen was 125 × 12.7 × 5 mm^3^ in dimension, with a V-notch 2.5 mm deep and a 45° angle.

## 3. Results and Discussions

### 3.1. Differential Scanning Calorimetry (DSC) Test Result

**Figure 5:**
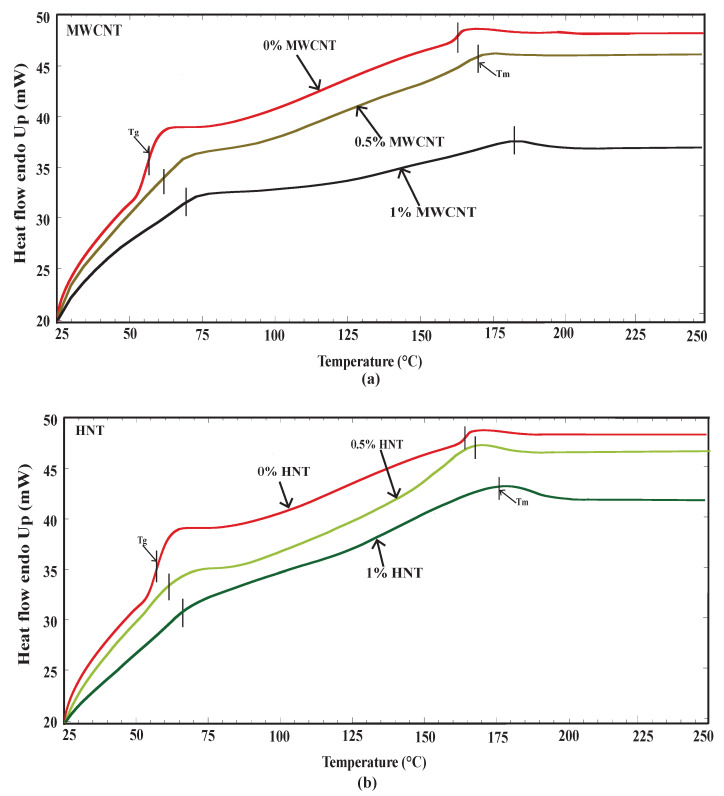
(**a**) DSC results for pure SMPU and SMPU reinforced with 0.5 and 1 wt% of MWCNTs and (**b**) DSC results for pure SMPU and SMPU reinforced with 0.5 and 1 wt% of HNTs.

The Tg and Tm of pure SMPU and specimens reinforced with MWCNTs and HNTs in important zones were evaluated accurately and peak values were identified. Figure 5 shows the heat flow in the sample during heating as a function of temperature while performing the DSC analysis. The corresponding Tg and Tm are marked with a small vertical line. From Figure 5a, the glass transition temperatures of 56.5, 63, and 69 °C and melting temperatures of 163, 171 and 182 °C can be observed for pure SMPU, 0.5 and 1 wt% MWCNTs-reinforced specimens. Further, for pure SMP, similarly, from Figure 5b, the glass transition temperatures of 62 and 66 °C and melting temperatures of 168 and 177 can be observed for 0.5 and 1 wt% HNTs-reinforced specimens. An increase in glass transition and melting temperatures can be observed with an increase in wt% of reinforcements. Specimens reinforced with HNTs exhibited a little lower glass transition temperature than specimens reinforced with MWCNTs. The obtained results are in line with those of [25,27,28].

### 3.2. Tensile Test Results

**Figure 6:**
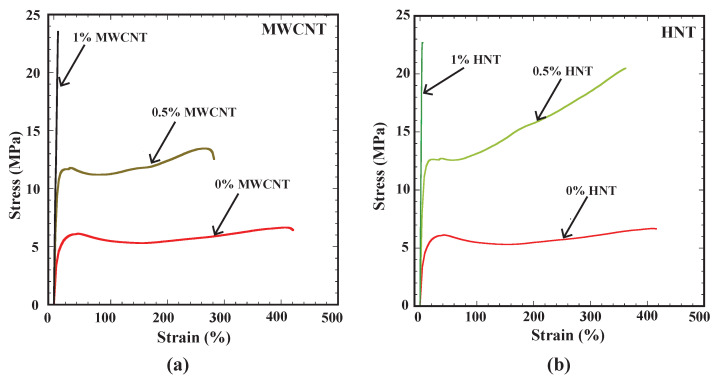
(**a**) Engineering stress vs. strain (%) for SMPU and MWCNTs composite specimens, (**b**) engineering stress vs. strain (%) for SMPU and HNTs composite specimens.

Figure 6a,b show the tensile test results (engineering stress vs. % of engineering strain) for two types of reinforcements, MWCNTs and HNTs, for corresponding 0 wt%, 0.5 wt% and 1 wt% in SMPU. Table 1 shows the tensile strength and percentage of elongation values for all considered cases. From Figure 6a,b, one can observe that in both cases of reinforcements (MWCNTs and HNTs), the tensile strength increased and the percentage of elongation reduced with an increase in the weight percentage of reinforcements. From Table 1, it is evident that at 0.5 wt%, HNTs gave a higher tensile strength than that of MWCNTs. Whereas at 1 wt%, MWCNTs gave a higher tensile strength than HNTs did. Further, one can understand that for HNTs, there was only a 1.3% increase in tensile strength while increasing the reinforcement from 0.5 to a 1 wt%. On the other hand, there was a 73% increase in tensile strength for MWCNTs for an increase in weight percentage from 0.5 to 1 wt%. From Table 1, it is understandable that pure SMPU specimen(s) exhibited the highest elongation percentage. At 0.5 wt%, HNTs exhibited a higher elongation percentage than specimens reinforced with MWCNTs. At 1 wt%, the elongation percentage was similar to both MWCNTs- and HNTs-reinforced specimens. Overall, Figure 6 and Table 1 convey that the highest tensile strength (23.5 MPa) was observed for specimens reinforced with 1% MWCNTs with a corresponding elongation percentage of 7.23. The increase in tensile strength was almost negligible for samples reinforced with HNTs with corresponding weight percentages of 0.5 and 1%. The obtained tensile results are in line with those of [25,29,30,31].

### 3.3. Flexural Test Results

**Figure 7:**
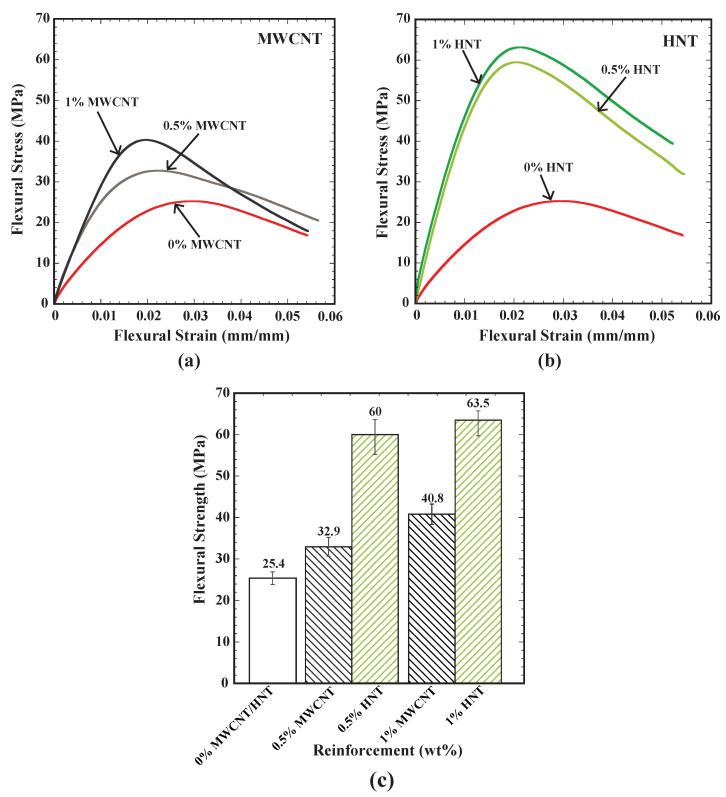
(**a**) Flexural stress vs. flexural strain for SMPU and MWCNTs composite specimens, (**b**) flexural stress vs. flexural strain for SMPU and HNTs composite specimens and (**c**) flexural strength for all considered specimens.

Figure 7 shows the flexural behaviour of SMPU specimens reinforced with MWCNTs and HNTs, for corresponding 0 wt%, 0.5 wt% and 1 wt% of reinforcement. Several interesting observations can be made from the figure: (i) the flexural strength increased with an increase in weight percentage of reinforcement for both MWCNTs and HNTs; (ii) for a given weight percentage of reinforcement, specimens reinforced with HNTs exhibited a higher flexural strength; (iii) no significant increase in flexural strength was observed for HNTs-reinforced samples with an increase in weight percentage from 0.5 to 1%; (iv) at 0.5 wt%, specimens reinforced with HNTs exhibited a 82% higher flexural strength than MWCNTs specimens; (v) at 1 wt%, specimens reinforced with HNTs exhibited a 56% higher flexural strength than MWCNT specimens; and (vi) among all considered cases, 1% HNTs-reinforced specimens gave the highest flexural strength of 63.5 MPa. Earlier studies [29] also showed that HNTs-reinforced specimens exhibited a higher flexural strength than MWCNTs-reinforced ones. The obtained results are in line with the literature results.

### 3.4. Flexural Test Results for Repeated Cycles

**Figure 8:**
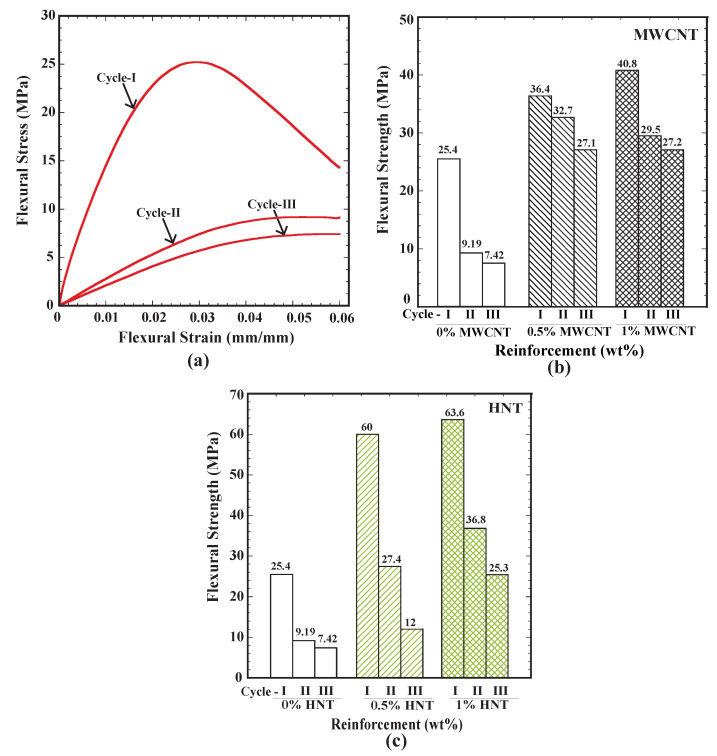
(**a**) Flexural stress vs. flexural strain for pure SMPU for three cycles, (**b**) flexural strength for three cycles of SMPU specimens reinforced with MWCNTs and (**c**) flexural strength for three cycles of SMPU specimens reinforced with HNTs.

For all the cases considered, after performing the flexural test, samples were heated to above the glass transition temperature to recover their original shape completely, and they were retested with a flexural test. This process was repeated for three cycles. Figure 8a shows the flexural stress vs. flexural strain curve for three cycles of flexural test for pure SMP. There was a significant reduction in flexural strength for each cycle. Figure 8b shows the variation of flexural strength for three cycles for MWCNTs-reinforced samples for the three weight percentages of 0, 0.5 and 1. For all weight percentages, there was a reduction in flexural strength for an increase in each repetitive cycle. The reduction in flexural strength from the first to the third cycle was highest in the case of 1 wt% MWCNTs-reinforced samples at 33.5%.

Further, there was a reduction of 28% in flexural strength from cycle 1 to cycle 2, whereas only an 8% reduction in flexural strength was observed from cycle 2 to cycle 3. Figure 8c shows the variation of the flexural strength for three cycles (for HNTs-reinforced samples) for the three weight percentages of 0, 0.5 and 1. As observed in MWCNTs samples, the flexural strength of HNTs-reinforced samples was reduced from one cycle to another. At 1% HNTs reinforcement, there was a reduction of 42% in flexural strength from the first cycle to the second cycle, whereas a 31% reduction in flexural strength could be observed from the second to the third cycle. HNTs-reinforced samples exhibited a higher loss of flexural strength from one cycle to another than MWCNTs-reinforced samples. Further, the reduction in flexural strength was lower as the number of cycles increased for all types of reinforcements considered in this study.

### 3.5. Shape Recovery Results

**Figure 9:**
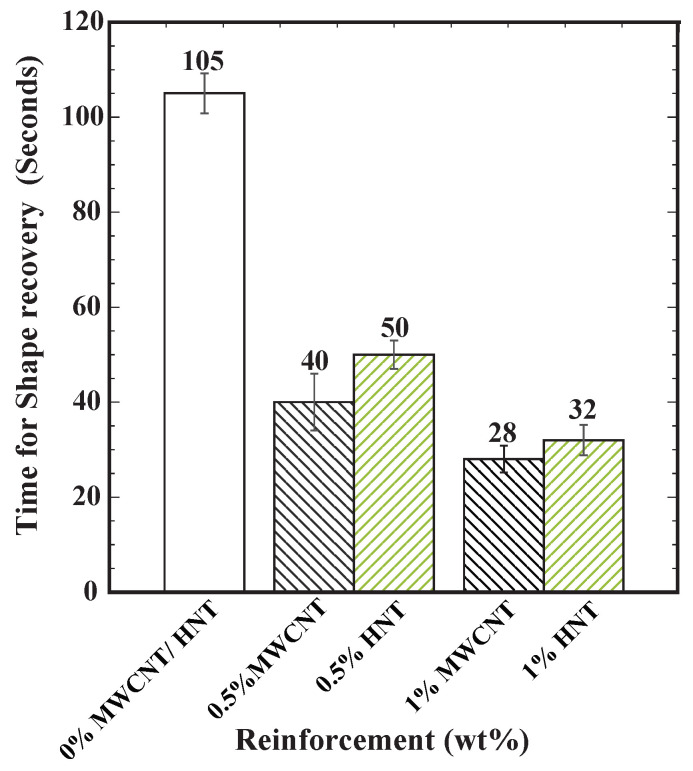
Shape recovery time (in seconds) for pure SMPU specimens and SMPU specimens reinforced with 0.5 and 1 wt% (by weight) MWCNTs and HNTs.

**Figure 10:**
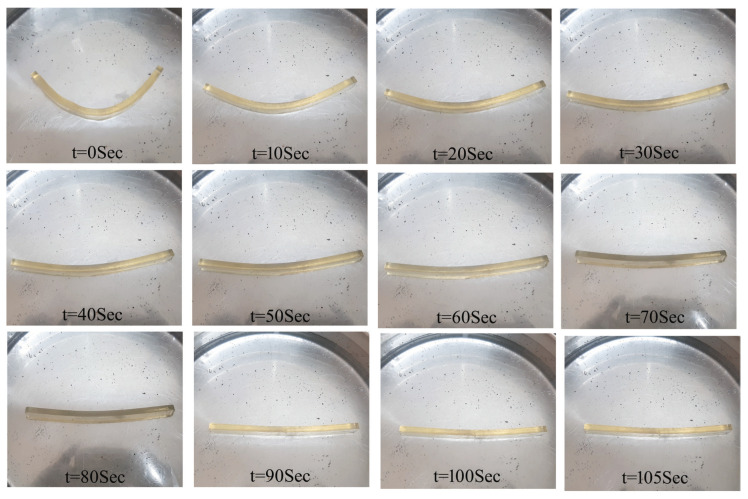
Shape recovery of a pure SMPU specimen at various time frames when placed in a water bath (at 80 °C).

**Figure 11:**
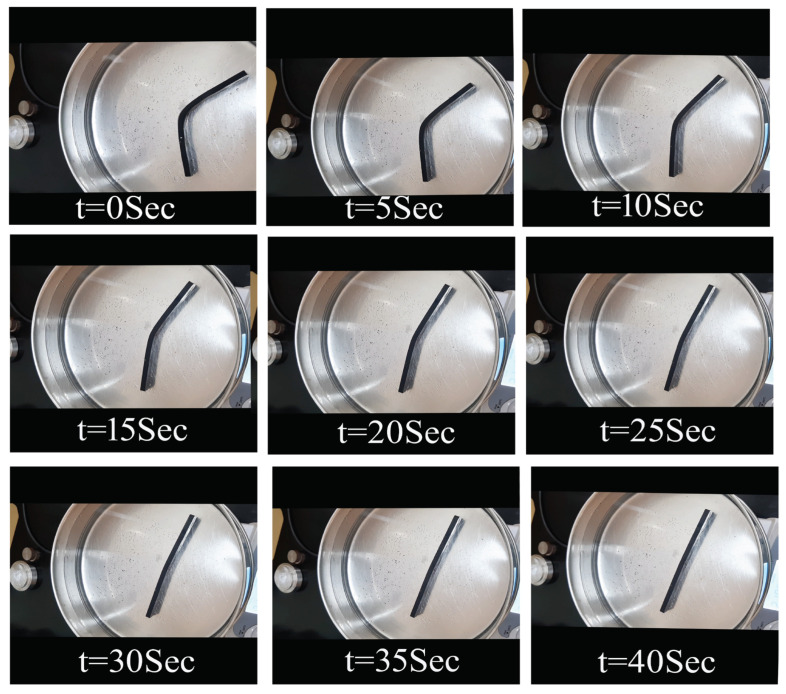
Shape recovery of pure SMPU and MWCNTs (0.5 wt%) composite specimens at various time frames when placed in a water bath (at 80 °C).

**Figure 12:**
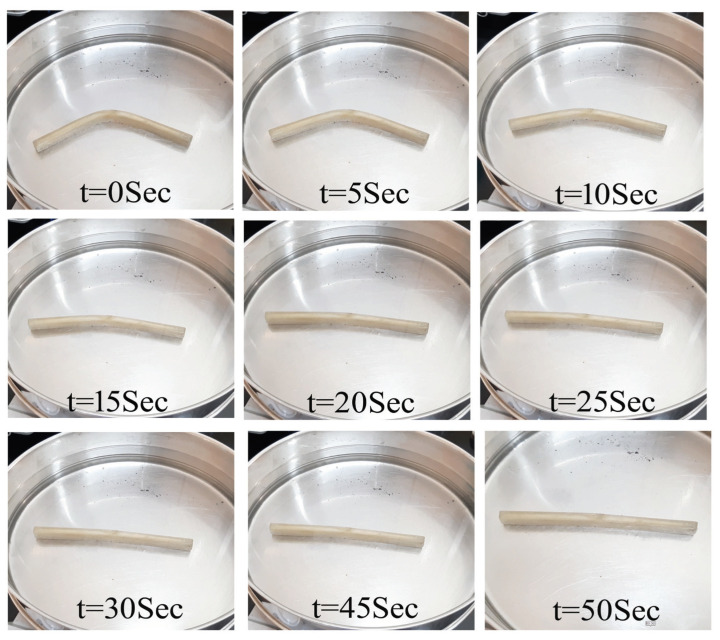
Shape recovery of SMPU and HNTs (0.5 wt%) composite specimens at various time frames when placed in a water bath (at 80 °C).

Figure 9 shows the time required for 100% shape recovery from the programmed temporary shape (deformed shape in flexural test). As the percentage of reinforcement increased, the shape recovery time reduced. MWCNTs-reinforced samples exhibited the lowest shape recovery time of 28 s at 1 wt% reinforcement in SMPU. Further, the shape recovery time was less in MWCNTs-reinforced samples when compared to HNTs-reinforced samples. Figure 10, Figure 11 and Figure 12 show the shape recovery at various time frames for pure SMP and SMP reinforced with 0.5 wt% MWCNTs and HNTs, respectively.

**Figure 13:**
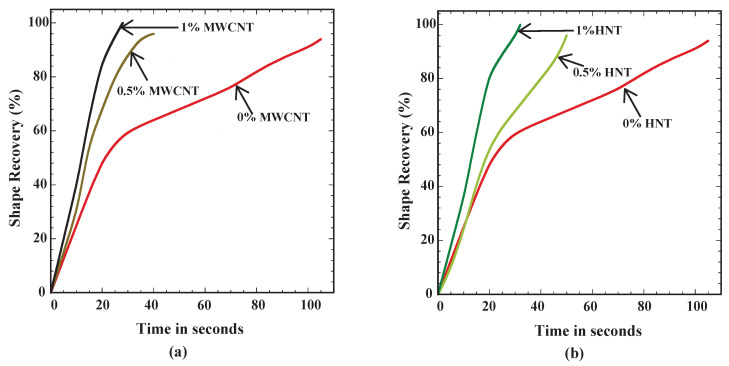
% of shape recovery as a function of time (in seconds) for (**a**) MWNCTs- and (**b**) HNTs-reinforced samples.

Figure 13 shows the % of shape recovery as a function of shape recovery time for pure SMPU and various reinforcement cases considered in the present study. The following observations can be made: (i) For pure SMPU, about 60% of the shape recovery could be observed during the first 30 s, and the remaining 40% of the recovery in 75 s (a sudden drop in slope could be observed after 30 s). This indicated that the shape recovery was rapid at the initial phase and slowed gradually. (ii) Higher slopes for 0.5 and 1% MWCNTs-reinforced specimens were clear evidence of a faster shape recovery for MWCNTs-reinforced specimens (see Figure 13a). (iii) Further, from Figure 13a, one can observe that at the end of 30 s, where pure SMPU recovered by 60%, the 0.5 wt% MWCNTs-reinforced specimen recovered by 90%, and the 1 wt% MWCNTs-reinforced specimen recovered by 100%. As the % of reinforcement increased, shape recovery was quicker.

Figure 13b shows the % of shape recovery as a function of shape recovery time for the pure SMPU specimen and 0.5 and 1 wt% HNT-reinforced specimens. One can observe the following from Figure 13b: (i) till 60% of the shape recovery, only a slight variation in recovery time could be observed for specimens with 0 and 0.5 wt% HNTs; (ii) for 1% HNTs-reinforced specimens, a drop in slope in the curve (which slows down the shape recovery) could be observed after a shape recovery of 80%. The higher shape recovery time for HNTs-reinforced specimens were evidence of a slower heat diffusion in HNTs samples compared to MWCNTs.

### 3.6. Impact Test Results

**Figure 14:**
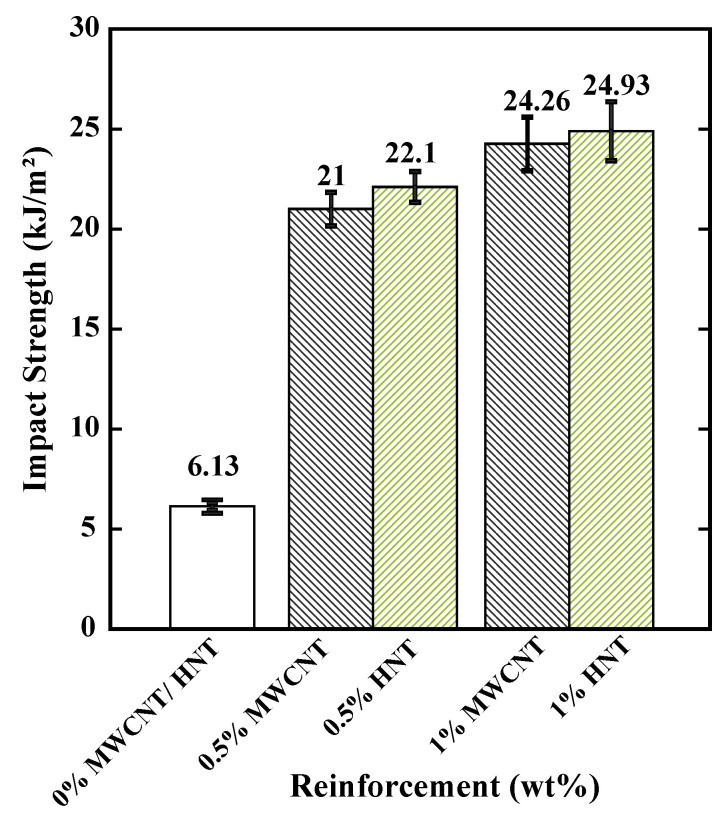
Impact strength obtained from Charpy test for pure SMPU, 0.5 and 1 wt% MWCNTs- and HNTs-reinforced samples.

Figure 14 shows the impact strength results of pure SMPU and specimens reinforced with 0.5 and 1 wt% of MWCNTs and HNTs. The following observations can be made from the figure: (i) the impact strength increased with an increase in the percentage of reinforcement (for both MWCNTs and HNTs); (ii) there existed only a 3–4% difference in impact strength for MWCNTs and HNTs at both 0.5 and 1 wt% reinforcements in SMP; (iii) an increase of 250% in impact energy can be observed while increasing the reinforcement from 0 to 0.5 wt%; and (iv) only a 14% increase in impact energy was observed while increasing the reinforcement from 0.5 to 1 wt%.

## 4. Conclusions

The following can be concluded from the experimental results of mechanical and shape recovery characteristics of SMPU reinforced with two types of reinforcements, MWCNTs and HNTs:

- The tensile strength and elongation percentage varied enormously with the addition of both MWCNTs and HNTs. The tensile strength increased significantly, and the percentage of elongation decreased with an increase in the weight percentage of reinforcement.
- SMPU reinforced with 0.5 wt% HNTs gave 22.4 MPa tensile strength, which was 5% less than the tensile strength of SMPU reinforced with 1 wt% MWCNTs.
- At a given weight percentage of reinforcement, SMPU reinforced with HNTs exhibited a higher flexural strength than SMPU reinforced with MWCNTs.
- There was a minor increase in tensile strength and flexural strength of SMPU reinforced with HNTs during the increase in weight percentage from 0.5 to 1.
- A significant drop in flexural strength could be observed while doing the flexural test for repeated cycles. The drop was higher in the case of SMPU reinforced with HNTs compared to SMPU reinforced with MWCNTs.
- At both considered weight percentages of reinforcements, there existed only a five-percent difference in impact strength of specimens reinforced with MWCNTs and HNTs.
- The time required for the shape recovery was less in the case of SMPU reinforced with MWCNTs (least at 1 wt%). Further, it was evident from the shape recovery curves that the shape recovery in specimens reinforced with MWCNTs was faster.

## Figures and Tables

**Table 1:** Tensile strength and % of elongation (obtained from tensile test) for pure SMPU, specimens reinforced with 0.5 and 1 wt% of MWCNTs and HNTs.

	Pure SMP	0.5 wt% Reinforcement		1 wt% Reinforcement	
Type of reinforcement		MWCNTs	HNTs	MWCNTs	HNTs
Tensile strength (MPa)	6.7	13.6	22.4	23.5	22.7
% of elongation	424	284	362	7.23	6

## Data Availability

Not applicable.

## Abbreviations

The following abbreviations are used in this manuscript:

SMPU	Shape-memory polyurethane
MWCNTs	Multiwalled carbon nanotubes
HNTs	Halloysite nanotubes
SMPs	Shape-memory polymers
SMPPs	Shape-memory polymer pellets
$\Theta_{ud}$	Angle of the sample in undeformed/permanent shape
$\Theta_{d}$	Angle of the sample in deformed/temporary shape
